# Work, life, and the gender effect: Perspectives of ACVIM Diplomates in 2017. Part 1—Specialty demographics and measures of professional achievement

**DOI:** 10.1111/jvim.15872

**Published:** 2020-08-17

**Authors:** Samantha L. Morello, Sara A. Colopy, Ruthanne Chun, Kevin A. Buhr

**Affiliations:** ^1^ Department of Surgical Sciences at University of Wisconsin‐Madison School of Veterinary Medicine Madison Wisconsin USA; ^2^ Department of Medical Sciences at University of Wisconsin‐Madison School of Veterinary Medicine Madison Wisconsin USA; ^3^ Department of Biomedical Informatics University of Wisconsin‐Madison, School of Medicine and Public Health Madison Wisconsin USA

**Keywords:** advancement, career, demographics, gender, salary, work‐life balance

## Abstract

**Background:**

Barriers to achieving work‐life balance, as well as gender‐based differences, exist in the male‐dominated surgical specialty in veterinary medicine. Similar information does not exist for the more feminized American College of Veterinary Internal Medicine (ACVIM).

**Hypothesis/Objectives:**

To provide data on the professional and personal lives of Diplomates of the ACVIM so as to help define the state of the specialty, including gender‐related differences, and identify areas requiring intervention to improve work‐life balance.

**Sample:**

A total of 896 surveys (781 completed) of Diplomates of the ACVIM, including cardiology, large animal internal medicine, neurology, oncology, and small animal internal medicine.

**Methods:**

An 82‐item online survey was distributed in February 2017 to ACVIM Diplomates via their respective ACVIM listserv. Participation was voluntary.

**Results:**

Thirty percent of the total ACVIM registered membership responded and 26% completed surveys; 25% were men and 75% were women. Specialists in academia worked significantly more hours, with larger numbers of diplomates per specialty section, and made less money compared with those in private practice. Women were less likely to report full‐time employment, practice ownership, or higher academic rank, and reported 20% lower income overall (after adjustment for relevant factors) as compared with men. Men and women differed in their subjective assessment of the effect of gender in the workplace. Eighty‐three percent of respondents were somewhat satisfied or better with their career.

**Conclusions and Clinical Importance:**

Specialization in the ACVIM is a satisfying and potentially profitable career. However, despite a highly feminized workforce, significant gender‐related imbalances are evident.

**Abbreviations**
ACVIM: American College of Veterinary Internal Medicine
ACVS: American College of Veterinary Surgeons
AVMA: American Veterinary Medical Association
LAIM: large animal internal medicine
SAIM: small animal internal medicine

## 1 INTRODUCTION

Since 1980, the American College of Veterinary Internal Medicine (ACVIM) has trained and promoted the fields of oncology, neurology, and cardiology, in addition to large and small animal internal medicine (SAIM). The ACVIM is the largest specialty organization recognized by the American Veterinary Medical Association (AVMA). Specialization is an attractive option for many reasons including in‐depth exploration of specific clinical interests, opportunities to pursue teaching, research or both in academia, and employment in a potentially more financially rewarding private referral setting. Despite these benefits, it is unclear what role specialization plays in affecting success, flexibility, or balance with life outside of work.

For most veterinarians, the relationship between career success and a happy personal life is not necessarily linear. Recently, a survey of Diplomates of the American College of Veterinary Surgeons (ACVS) evaluated the professional landscape and elements of personal life to provide an objective data set.^1^, ^2^ The study identified gender‐related gaps in compensation, academic advancement, and practice ownership, as well as variable perceptions about these points between male and female veterinary surgeons. Notable differences also were found between academic and private practice environments, and even larger disparities were found between those practicing on large vs small animals. These results prompted us to seek similar data about specialists in the ACVIM.

Feminization of labor markets has been shown to negatively affect status and average compensation of a profession, and data suggest that veterinary medicine may follow this trend.^3^, ^4^, ^5^ As of 2009, female veterinarians outnumbered their male counterparts in the work force, yet in 2011 the AVMA reported a 21% gap in median income between female and male veterinarians in private practice.^6^ The majority of ACVS Diplomates are men,^7^ and male surgeons have 18% higher annual incomes and are more likely to advance into prestigious professional roles, as well as be married and have children, compared with female surgeons.^1^, ^2^ In 2017, the ACVIM and its respective specialties were predominantly female (personal communication, ACVIM). It is unknown if the gender differences noted among ACVS Diplomates also would occur in a field where women predominate or in a specialty that may impose different stressors. Consideration of the effects of gender on a specialty organization that already has a marked female majority could serve as a valuable lens for other areas of the veterinary profession, and may help inform changes in policy or procedure. For specialty practice to remain rewarding and appealing to a younger generation of veterinarians, aspects of the profession may need to adjust to meet the needs and desires of a changing group.

Our objective was to collect data about the professional and personal lives of ACVIM Diplomates through an anonymous online survey. The aims were to examine these findings with respect to relevant covariates, summarize the current status of ACVIM specialists, and identify specific factors that may influence a successful and satisfying career.

## 2 MATERIALS AND METHODS

### 2.1 Study population

The study group consisted of Diplomates of the ACVIM in good standing as of February 2017. At the time of the survey, ACVIM membership was as follows: large animal internal medicine (LAIM, 625 members), SAIM (1369 members), cardiology (273 members), neurology (294 members), and oncology (393 members) with proportions of ACVIM Diplomates by gender as follows: overall—37.5% male, 62.5% female; LAIM—36% male, 64% female; SAIM—36% male, 64% female; cardiology—46% male, 54% female; neurology—45% male, 55% female; oncology—31% male, 69% female (personal communication, ACVIM). Diplomates were contacted via 5 specialty listservs. Individual email addresses were not obtained, and the number of registered diplomates with active email addresses subscribed to the listservs was unknown. The Institutional Review Board at the University of Wisconsin‐Madison waived formal review of the protocol in accordance with Federal Regulation 45 CFR 46.102(d).

### 2.2 The survey

An 82‐item questionnaire (Supporting Information Item 1) was created and administered using an online platform (Qualtrics©, Provo, Utah) to evaluate: (a) respondent demographics (eg, age, gender, location, ethnicity); (b) professional logistics (eg, type of practice, rank or title, number of hours worked, on‐call duty); (c) compensation; and (d) information about the respondents' personal life and family (eg, marital status, presence and number of children, childcare arrangements, age at first child). Questions designed to elicit subjective data were asked regarding: (a) the effects of career and family on each other; (b) career satisfaction; and (c) any effect of gender on aspects of professional life. The survey link was disseminated by email (Supporting Information Item 2). All data collected were anonymous and automatically entered into a computerized database for analysis. Responses were collected over the course of 1 month, and Diplomates were prompted for participation 3 times by email.

### 2.3 Study analysis

Response counts and percentages (of nonmissing responses) were calculated. For group comparisons of categorical responses, *P* values were calculated using Chi‐squared tests. For continuous variables with categorical coding (eg, age), *P* values were calculated using simple linear regression on category midpoints. For personal income, which was subject to right censoring at $400K, *P* values for simple group comparisons were calculated using Wilcoxon rank‐sum or Kruskal‐Wallis tests, and regression models with adjustments for covariates were fitted using parametric survival analysis under a Weibull distribution assumption. Although such analysis is most commonly used for survival data, it is also appropriate for other types of data subject to right censoring. Regression models included covariate adjustments for gender, age, race, geographic region of employment, employment status, year in which diplomate status was obtained, specialty, an academic rank or title score (in which each promotion level was assigned 2 additional points, and tenure track positions were given 1 point), and private practice ownership status. All statistical analyses were performed using R version 3.6.3 (*R Core Team*, R Foundation of Statistical Computing, Vienna, Austria, 2020).

## 3 RESULTS

### 3.1 Response demographics

A total of 896 individuals responded with 781 (87.2%) finishing the survey. All results described below are based on analyses of finished surveys. A sensitivity analysis of data was conducted that included incomplete surveys, and results were similar to results presented here. Across all survey responses analyzed, the percentage of missing responses was ≤5%.

Response by specialty was: SAIM, 294 (37.8%); LAIM, 175 (22.5%; equine, farm animal, or mixed); cardiology, 84 (10.8%); neurology, 95 (12.2%); and oncology, 130 (16.7%) with 3 not providing a specialty. For LAIM, 112 reported residency training in mixed large animal medicine, and 54 and 9 trained exclusively in equine or farm animal medicine, respectively.

**FIGURE 1:**
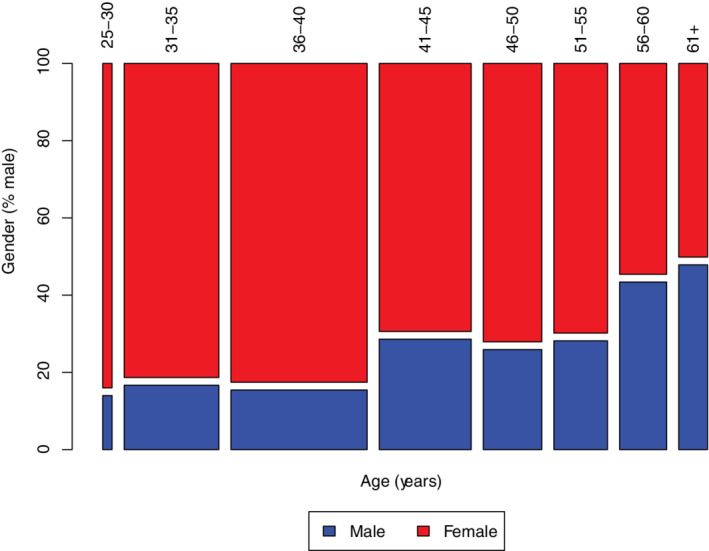
Mosaic plot showing the distribution of men (blue bars) and women (red bars) by age category for ACVIM Diplomates who responded to questions regarding gender and age (n = 775). The area of each bar is proportional to number of respondents in that category

Of those providing information regarding gender (n = 776), 25.3% (n = 196) identified as male, 74.6% (n = 579) as female, and 0.1% (n = 1) as other. For each specialty, the percentages of male : female : other respondents were: SAIM, 28.7/71.3/0; LAIM, 20.7/78.7/0.6; cardiology, 29.8/70.2/0.0; neurology, 30.5/69.5/0; and oncology, 16.9/83.1/0. Although demographics for listserv subscribers could not be determined, based on comparison with college demographics, women were more likely to respond than men (odds ratio = 2.13; *P* < .001). Of those reporting race or ethnicity, the majority were white (n = 70, 91.4%); Hispanic/Latino (n = 26, 3.4%), Asian/Pacific islander (n = 18, 2.3%), black/African American (n = 6, 0.8%), Native American/American Indian (n = 2, 0.3%), or other (n = 14, 1.8%). The median age for all participants was 41‐45 years; median age for men was 46 to 50 years and for women was 36 to 40 years (*P <* .001; Figure 1). Most Diplomates reported living in the United States (n = 647, 83.3%), followed by Canada (n = 50, 6.4%), Europe (n = 49, 6.3%), Australia/New Zealand (n = 24, 3.1%), Asia (n = 6, 0.8%), and Africa (n = 1, 0.1%). Within the United States, 26% resided in the West (n = 166), 23% in each of the Northeast and Southeast (n = 150, n = 149), 20% in the Midwest (n = 129), 7% in the Southwest (n = 47), and the remaining 1% in Hawaii and Alaska (n = 3; see Supporting Information Item 1).

In addition to a veterinary medical degree and board certification by the ACVIM, the following degrees also were reported: Master of Science (MS), 35.6% (n = 256); Doctor of Philosophy (PhD), 16.7% (n = 120); additional board certification, 12.7% (n = 91); Master of Business Administration (MBA), 1.1% (n = 8); and other, 13.2% (n = 95).

### 3.2 Professional demographics

The median year when respondents achieved diplomate status was 2008 (n = 768). The median number of jobs held since residency was 2 (n = 777). At the time of survey, employment status was part‐time for 8.6% (n = 67), unemployed for 0.6% (n = 5), and full‐time for 90.7% (n = 703), with no differences by specialty. Respondents in private practice were more likely to report part‐time employment than were those in academia (9.6% vs 6%, *P* < .001). Women were less likely than men to report full‐time employment (n = 514, 88.8% vs n = 186, 94.9%; *P* = .01). Part‐time employment was not defined in the survey; 8.4% of respondents who reported working full‐time also reported working <40 hours/wk (n = 59) and 22.4% of those working part‐time also reported working ≥40 hours/wk (n = 15), suggesting that interpretation was not strictly based on hours worked per week.

Ninety‐one percent (n = 708) responded that they currently were practicing clinical veterinary medicine. This proportion was lower for LAIM (82.8%; *P* < .001) compared with SAIM (88.7%) and cardiology, neurology, and oncology (98.4%). When queried whether their scope of practice was limited to the specialty field in which they trained, respondents trained in cardiology (n = 83, 98.8%), neurology (n = 92, 97.9%), and oncology (n = 128, 98.5%) were more likely to respond “yes” than those trained in SAIM (n = 257, 88.3%) or LAIM (n = 103, 59.2%, *P* < .001). Most (n = 727, 93.6%) were practicing within the species area of emphasis for which they trained during residency, but this varied by specialty (LAIM, 82.9%; SAIM, 93.2%; cardiology, neurology, and oncology, 99.7%, *P* < .001).

At the time of survey, 33.7% of respondents were employed in academia, 58.1% in private practice, and 8.3% in another setting (Table 1). The LAIM respondents were most likely to be employed in academia (60.3%) whereas SAIM, cardiology, neurology, and oncology respondents were most likely to be in private practice (62.8% for SAIM, 70.5% for others). Employment by practice type (academia, private practice, and other) did not differ by gender (*P* = .99).

**TABLE 1:** Area of employment by specialty

	Academia	PP/GP	Industry	Other
SAIM	25.3% (74)	62.8% (184)	6.8% (20)	5.1% (15)
LAIM	60.3% (105)	28.2% (49)	4.6% (8)	6.9%(12)
Equine, only	46.3% (25)	44.4% (24)	3.7% (2)	5.6% (3)
Farm animal, only	88.9% (8)	0	11.1% (1)	0
Mixed, equine and farm animal	64.9% (72)	22.5% (25)	4.5% (5)	8.1% (9)
Cardiology	31.0% (26)	65.5% (55)	1.2% (1)	2.4% (2)
Neurology	25.5% (23)	71.3% (67)	0	3.2% (3)
Oncology	24.6% (32)	73.1% (95)	0	2.3% (3)

*Note:* Numbers in parentheses correspond to the number of respondents within that category. Percentage is given as a proportion of the overall specialty (ie, 26.3% of SAIM Diplomates are employed in academia).

Abbreviations: GP, general practice; LAIM, large animal internal medicine; PP, private practice; SAIM, small animal internal medicine.

Respondents were asked to report the total number of ACVIM Diplomates in the specialty section in which they worked within their own practice or institution. Although not a direct measure of practice size, this question was used to determine the average size of specialty groups in different practice settings. Overall, practice size was larger by this measure in academia than in private practice (*P* < .001; Table 2).

**TABLE 2:** Mean, median number of Diplomates employed in each specialty department

	SAIM	LAIM	Cardiology	Neurology	Oncology
Academia	6.2, 6	5.0, 5	2.2,2	2.9, 3	4.5, 4
Private Practice	2.8, 2	1.3, 1	2.1, 1	2.3, 2	2.6, 2

Abbreviations: LAIM, large animal internal medicine; SAIM, small animal internal medicine.

Respondents were asked to estimate the average number of hours worked per week, and the average number of on call shifts covered per month; results were stratified by specialty and practice type (Tables 3 and 4). For all specialty groups, the median number of hours worked per week in private practice was 40 to 49 hours vs 50 to 59 hours in academia. Overall, respondents in private practice reported working fewer hours than those in academia (5.2 fewer hours/wk, *P* < .001). There was some evidence that male diplomates worked more hours than female diplomates in private practice (estimated 2.9 more hours, *P* = .004); this difference may have been related to differences in practice ownership by gender (see later).

**TABLE 3:** Semiquantitative distribution of hours worked per week (self‐reported) for ACVIM Diplomates in private practice and academia (n = 770)

# hours/wk	<40	40‐49	50‐59	60+	Median (h/week)	Est. mean (h/wk)
*SAIM*						
Academia	4.1%	24.3%	39.2%	32.4%	50‐59	55.0
PP	14.7%	42.9%	28.8%	13.6%	40‐49	49.1
*LAIM*						
Academia	8.7%	30.8%	41.3%	19.2%	50‐59	52.1
PP	18.4%	36.7%	22.4%	22.4%	40‐49	49.9
*Cardiology*						
Academia	0.0%	42.3%	34.6%	23.1%	50‐59	53.1
PP	32.7%	34.5%	23.6%	9.1%	40‐49	45.9
*Neurology*						
Academia	4.2%	20.8%	54.2%	20.8%	50‐59	54.2
PP	10.4%	41.8%	28.4%	19.4%	40‐49	50.7
*Oncology*						
Academia	6.2%	25.0%	40.6%	28.1%	50‐59	54.1
PP	20.0%	58.9%	15.8%	5.3%	40‐49	45.6

*Note:* Rows are categorized by specialty, and subcategorized by employment setting (academia vs PP). Percentages correlate to the proportion of individuals responding in each employment setting. The estimated mean is calculated (est. mean) for each subcategory.

Abbreviations: LAIM, large animal internal medicine; PP, private practice; SAIM, small animal internal medicine.

**TABLE 4:** Semiquantitative distribution of average on call shifts per month (self‐reported) for ACVIM Diplomates in academia and private practice (n = 741)

# nights/mo.	0	1‐3	4‐7	7‐10	10‐14	15+	Median (shifts/mo.)	Est. mean (shifts/mo.)
*SAIM*								
Academia	18.5%	20.0%	21.5%	13.8%	21.5%	4.6%	**4‐7**	**6.0**
PP	28.6%	19.8%	13.7%	10.4%	6.0%	21.4%	**4‐7**	**6.3**
*LAIM*								
Academia	18.0%	18.0%	18.0%	15.7%	20.2%	10.1%	**4‐7**	**6.7**
PP	10.2%	10.2%	20.4%	12.2%	16.3%	30.6%	**7‐10**	**9.4**
*Cardiology*								
Academia	3.8%	23.1%	19.2%	11.5%	26.9%	15.4%	**7‐10**	**8.2**
PP	47.3%	16.4%	5.5%	3.6%	1.8%	25.5%	**1‐3**	**5.4**
*Neurology*								
Academia	16.7%	16.7%	8.3%	12.5%	20.8%	25.0%	**7‐10**	**8.5**
PP	12.1%	13.6%	22.7%	6.1%	18.2%	27.3%	**7‐10**	**8.8**
*Oncology*								
Academia	25.0%	37.5%	9.4%	9.4%	9.4%	9.4%	**1‐3**	**4.7**
PP	55.8%	12.6%	4.2%	6.3%	2.1%	18.9%	**0**	**4.4**

*Note:* Rows are categorized by specialty, and subcategorized by employment setting (academia vs PP). Percentages correlate to the proportion of individuals responding in each employment setting. The estimated mean is calculated (est. mean) for each subcategory.

Abbreviations: LAIM, large animal internal medicine; PP, private practice; SAIM, small animal internal medicine.

The median number of nights spent on call for SAIM Diplomates was 4 to 7 per month for both private practice and academia. This number was significantly higher for LAIM respondents in private practice (7‐10 per month) compared with academia (4‐7 per month, *P* = .008). The LAIM Diplomates in private practice also represented the highest proportion of specialists with ≥15 on call shifts per month (30.6%). Oncologists spent the least amount of time on call overall (*P* < .001). No gender difference was found in time spent on call.

### 3.3 Objective measures of professional success

Income was used as a quantitative measure of success. Respondents provided their personal annual pretax income in thousands of dollars if their salary was <$400K or indicated that their salary was ≥$400K per year. Overall median income for ACVIM respondents was significantly lower in academia (n = 258, $120K) than in private practice (n = 435, $170K; *P* < .001); this difference was significant for all specialties, with the exception of LAIM, for which incomes were equivalent across practice settings (Table 5).

**TABLE 5:** Median income (in thousands, K) for ACVIM Diplomates by specialty, practice setting, and gender

	Men	Women	Overall
*SAIM*			
Academia	$132K; 0% (0/23)	$120K; 0% (0/49)	**$125K; 0% (0/72)**
PP	$194K; 10.4% (5/48)	$162K; 0% (0/129)	**$170K; 2.8% (5/177)**
*LAIM*			
Academia	$137K; 0% (0/23)	$108K; 0% (0/81)	**$114K; 0% (0/105)**
PP	$150K; 0% (0/7)	$101K; 0% (0/39)	**$110K; 0% (0/46)**
*Cardiology*			
Academia	$139K; 0% (0/8)	$120K; 0% (0/18)	**$121K; 0% (0/26)**
PP	$264K; 31.2% (5/16)	$157K; 0% (0/38)	**$166K; 9.3% (5/54)**
*Neurology*			
Academia	$128K; 0% (0/4)	$123K; 0% (0/19)	**$123K; 0% (0/23)**
PP	$252K; 13.0% (3/23)	$180K; 0% (0/43)	**$200K; 4.5% (3/66)**
*Oncology*			
Academia	$145K; 0% (0/6)	$126K; 0% (0/26)	**$129K; 0% (0/32)**
PP	$230K; 0% (0/15)	$169K; 2.6% (2/77)	**$180K; 2.2% (2/92)**
*Industry*	$167K; 0% (0/9)	$120K; 0% (0/19)	**$139K; 0% (0/28)**
*Other (incl. military)*	$113K; 0% (0/6)	$100K; 0% (0/28)	**$102K; 0% (0/35)**

*Note:* The percentage and number of individuals making more than $400K/y is reported for each category.

Abbreviations: LAIM, large animal internal medicine; PP, private practice; SAIM, small animal internal medicine.

**FIGURE 2:**
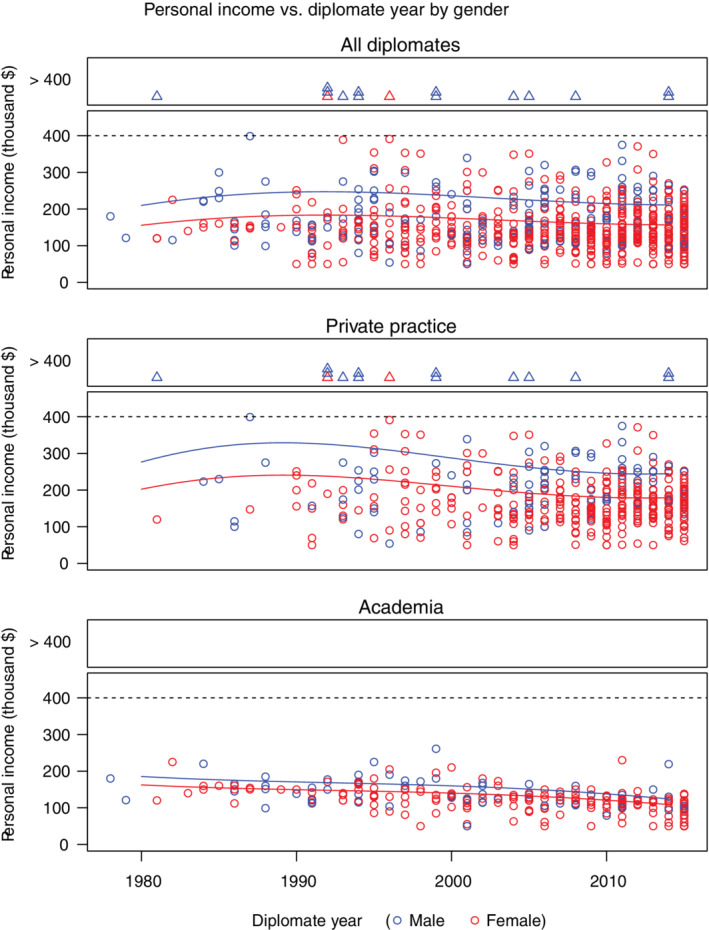
Personal income by diplomate year for men (blue) and women (red) in the ACVIM who responded to questions regarding income and gender overall (n = 748), and for only those in private practice (n = 427) or academia (n = 256). Respondents were asked to report their income on a sliding scale to a maximum of $400 000. Within each panel, the bottom subpanel shows a scatterplot of incomes <$400 000 (each symbol is equal to one respondent) while the top subpanel represents respondents with incomes ≥$400 000. The smoothed curves reflect parametric fit estimating mean salary for both genders by year of receiving diplomate status. Overall, annual income for men was 20% higher than for women (*P* < .001). This gender difference was greater in private practice (29%; *P* < .001) and less in academia (9%; *P* = .05)

Men reported significantly higher incomes than did women (median $170K vs $135K, *P* < .001). Reported income for men was on average 20% higher (95% confidence interval [CI], 14%‐27%; *P* < .001) after adjusting for the relevant covariates of age, race, region, employment status, year of diplomate status, specialty species, practice type, academic title, and practice ownership status. This difference was evident in both academia and private practice (Figure 2). After adjusting for these covariates, the difference was less marked in academia (9% gap; 95% CI, 2%‐17%; *P* = .05) than in private practice (29% gap; 95% CI, 19%‐40%; *P* < .001).

As another quantitative measure of career success, respondents in private practice were asked to categorize their employment status as associates, partners with <50% ownership of the practice, partners with ≥50% ownership, or as locums or other types of employee. Overall, 71% were associates (n = 318), and 13% (n = 58) and 9.6% (n = 43) described themselves as ≥50% and <50% owners, respectively. Men were significantly more likely than women to participate in practice ownership (n = 43, 38% vs n = 58, 17%, *P <* .001). There were differences in ownership by specialty (*P* = .001); LAIM Diplomates were most likely to participate in practice ownership of some kind (41%) followed by cardiologists (35%), SAIM Diplomates (21%), and neurologists and oncologists (16% and 13%, respectively).

For specialists in private practice, income was evaluated as a function of job title. The LAIM respondents were considered separately because they reported significantly lower income than did other specialists. Associates in equine, food animal, or large animal private practice reported a median annual income of $101K, which was significantly lower than both <50% owners (median, $125K) and >50% owners (median, $121K). Other specialties (SAIM, cardiology, neurology, oncology) were considered as a single group. Within this cohort, associates reported a lower median income ($170K) as compared with those who were <50% owners ($232K) or >50% owners ($201K). The relationship between minority vs majority ownership and salary was likely confounded by practice size; those who reported being <50% owners also were more likely to report larger numbers of diplomates in the specialty section to which they belonged.

In private practice, income is often a function of production revenue and was evaluated as a function of time spent working. Among specialists working full‐time in private practice, those who reported working the fewest hours (<40/wk) had the lowest median earnings ($159K/y) whereas those who worked >60 hours/wk had the highest ($200K/y); each additional 10 hours/wk of work was associated with an 8.5% increase in income (*P* < .001).

Diplomates were asked about their roles as earners in a household. Among respondents who listed themselves as single (never married, separated, divorced, widow, or widower), 93% (n = 165) described themselves as the primary earner. Among respondents listed as married or in a domestic partnership, 54% (n = 323) indicated they were the primary earners and 26% (n = 153) reported that their income was relatively equivalent to another member of the household. The married/domestic partnership group was further evaluated within the context of gender; different proportions of women (n = 198, 47%) and men (n = 123, 73%) identified themselves as the primary household earner (*P* < .001).

**FIGURE 3:**
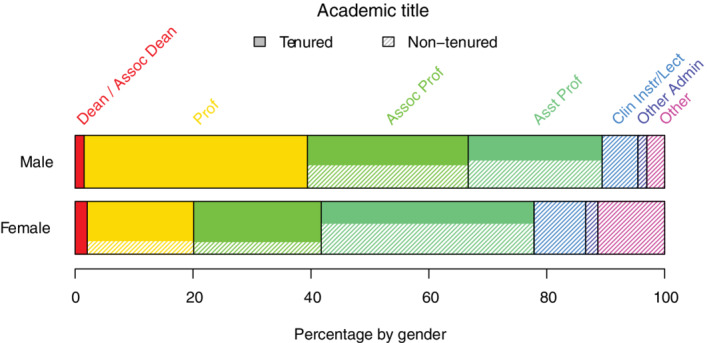
Strip plots of academic title (from left to right; dean/associate dean [red, far left], professor [yellow], associate professor [lime green], assistant professor [sea green], clinical instructor or lecturer [blue], other administration [purple], or “other” [pink, far right]) by gender for those in academia (n = 260). Each title within the professor track is separated into tenure‐track (solid fill) and nontenure track (shaded fill) positions; the proportion of those in each track is represented by the filled area of the bar

Those employed in an academic setting were asked to define their job title based on rank (instructor, assistant professor, associate professor, full professor, dean or associate dean, or other administrative role) and track (nontenure vs tenure) as another indirect measure of professional success (Figure 3). Thirty‐eight percent of men (n = 25) reported full professorship status compared to 18% of women (n = 35). One man and 4 women reported being in senior administrative (dean or associate dean) positions. Of those listed as assistant, associate, or full professors, 72% of men (n = 42) and 59% of women (n = 87) were either tenured or in tenure‐track positions. After assigning a score to title based on rank (each promotion level worth 2 points) and track (tenure track worth 1 point), men were more likely to hold a more prestigious title than women by a factor of 1.3 points (*P* < .001). This difference was no longer significant after adjustment for age, employment status (full‐ or part‐time), and year of becoming a diplomate.

### 3.4 Subjective assessments of professional issues

As an indirect measure of work‐life balance, diplomates were asked about their ability to balance the demands of career with time available for other pursuits. Overall, 81% (n = 624) of respondents agreed (36% strongly, 45% somewhat) that their career negatively affected their ability to pursue interests outside of work. Women were slightly more likely than men to agree with this statement (n = 478, 83% vs n = 144, 73%, *P* = .01).

From a list of 9 factors that played a role when choosing professional positions, Diplomates ranked passion for the job as the top influential factor. Quality of work‐life balance and location were ranked equally as the second most influential factors, followed by financial compensation. Emergency responsibilities and ability to do research were ranked as the least influential factors.

**FIGURE 4:**
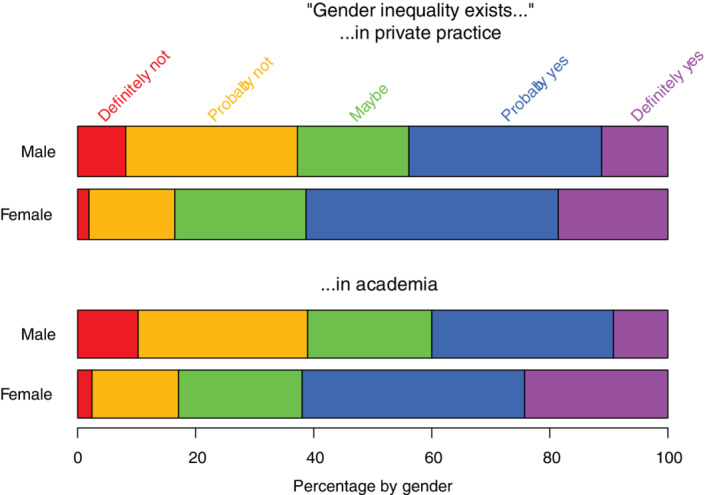
Strip plots showing the distribution of responses, by gender, to the questions “Apart from your own experience, do you feel that gender inequality exists between male and female surgeons in private practice?” (n = 767) and “in academic practice?” (n = 768). Response options, from left to right, included definitely not (red, far left), probably not (orange), maybe (green), probably yes (blue), and definitely yes (purple, far right)

Participants were asked, apart from their own experiences, whether they perceived gender inequality in their workplace (Figure 4). Women were more likely than men to indicate that gender inequality either probably or definitely existed in private practice (n = 350, 61% vs n = 86, 44%, respectively, *P* < .001) and in academia (n = 355, 62% vs n = 78, 40%, respectively; *P* < .001).

**FIGURE 5:**
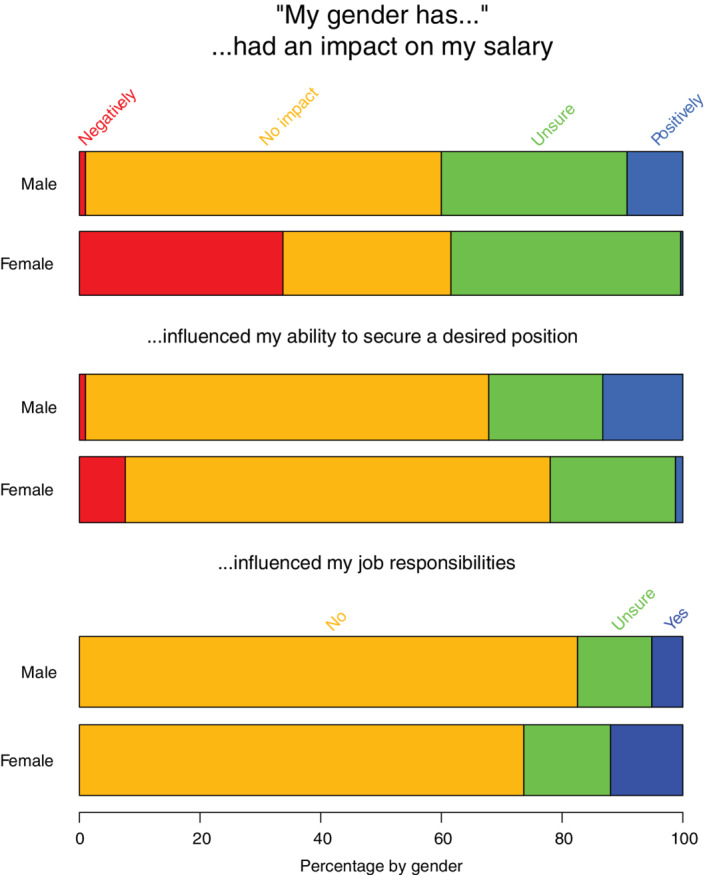
Strip plots of the perceived impact of gender, by male and female Diplomates responding to the survey, on salary (n = 773), the ability to secure a desired position (n = 774), and whether gender had influenced their job responsibilities (n = 772). Response options regarding salary and ability to secure a desired position included, from left to right, negatively (red, far left), no impact (orange), unsure (green), and positively (blue, far right). Options for the question on job responsibilities included no (orange, left), unsure (green, middle), and yes (blue, right), but do not reflect whether any perceived influence was negative or positive

Respondents reported whether they believed gender impacted their salary, position, responsibilities, or client interactions (Figure 5). Thirty‐four percent (n = 195) of women believed their income was lower than that of similarly qualified male colleagues, whereas only 9% (n = 18) of men believed that their salary was higher than that of similarly qualified female colleagues. Twenty‐eight percent (n = 161) of women and 59% (n = 115) of men believed their salaries to be equal to those of similarly qualified colleagues of the opposite gender. Although most men and women felt gender had not influenced their ability to acquire or retain a desired job (n = 407, 70% female and n = 131, 67% male respondents), 13% (n = 26) of men vs only 1% (n = 7) of women felt their gender had had a positive impact (*P* < .001). Women were more likely than men (n = 86, 15% vs n = 4, 2%, *P* < .001) to indicate their gender had negatively affected their ability to be promoted. Relatively few women (n = 69, 12%) and men (n = 10, 5%) indicated that gender had influenced job responsibilities; a somewhat larger proportion indicated that gender had affected interaction with clients (n = 214, 37% of women and n = 51, 26% of men) in some way. Finally, more women (n = 270, 47%) than men (n = 37, 19%; *P* < .001) responded that they had received comments about their gender related to career performance, potential, or productivity.

The final area of subjective questioning was designed to measure the level of happiness diplomates felt in their career. Most respondents (n = 643, 83%) were at least somewhat satisfied with their career; only 14% (n = 106) were at least somewhat dissatisfied. When asked about their achievement of balance between satisfying career and satisfying personal life, 53% (n = 415) of all respondents agreed whereas 42% (n = 327) of respondents disagreed. There was no difference by gender or specialty.

## 4 DISCUSSION

Observations of the ACVIM, a specialty organization that has more women than men in its membership, can provide insight for other specialty organizations, and the veterinary profession at large, as those areas shift to a predominantly female workforce. An overall response rate to our survey could not be calculated, because listservs were used rather than individual email addresses. However, one‐third of the number of Diplomates registered with the ACVIM responded to the survey, and listservs were unlikely to reach every diplomate. Women were overrepresented among respondents compared with college‐wide demographics. Similarly, a nonresponse bias could not be calculated, owing to a lack of available demographic data for nonresponders. Although it is likely that a nonresponse bias exists, we were able to capture data on a substantial proportion of the college membership, with the specialties represented among respondents being representative of the overall college. The data provided here reflect the objective and subjective facts and opinions of those who responded to the survey, and this sample population may be used to provide an estimate that reflects the college as a whole.

The ACVIM is already predominantly female, and will continue to become more disproportionately so, because 84% of Diplomates under 40 years of age identified as women, including 92.5% of those within the oncology specialty. This finding of the youngest (< 40 years) cohort comprising mostly women is similar to an identical study conducted among ACVS Diplomates,^1^ currently a predominantly male organization. Although the veterinary medical workforce currently is reported to be >60% female, that percentage is still less than what is observed in the ACVIM.^8^ A comparison of the datasets from these colleges provides an opportunity to predict characteristics of an increasingly feminized workforce. Identifying the contrasts between these demographically distinct groups may delineate areas where the profession could adapt to help serve an increasingly female workforce.

Employment demographics of the ACVIM indicate a specialty career with an assortment of opportunities. Overall, 8.6% of ACVIM Diplomates reported part‐time employment; part‐time employment was highest for cardiologists, those in private practice, and women, and lowest for those in academia and for men. Although part‐time employment was not defined in the survey, fewer people indicated working part‐time than working <40 h/wk, which suggests other criteria for part‐time work such as benefits, job title, or an even greater reduction in working hours. The 2019 AVMA report on the market for veterinarians demonstrated that women are more likely than men to report negative underemployment (ie, the desire to work fewer hours).^8^ Increased opportunities for part‐time employment among ACVIM Diplomates may be a consequence of a highly feminized workforce. It is also possible that some or all of the specialties within the college are more conducive to part‐time work, and this factor may have contributed to attracting a higher proportion of women. Part‐time employment across all areas of veterinary medicine should be monitored for such trends to better understand and promote career satisfaction and work‐life balance.

Among small animal and large animal internists, 6.8% and 4.6%, respectively, reported industry employment, which is in contrast to 0.7% of ACVS Diplomates.^1^ The training provided in an internal medicine residency may present specific value to certain industries. Among LAIM and SAIM, approximately 83% and 89% reported practicing clinical medicine (vs practicing outside of a strictly clinical realm), again highlighting other opportunities for ACVIM specialists. With respect to LAIM, industry may offer additional career prospects in the face of a relative paucity of job openings. Although personal or professional motivations are impossible to quantify, this information is useful information for diplomates considering a career change or for attracting those with an interest in industry to pursuing ACVIM board certification.

The majority of Diplomates specializing in SAIM, cardiology, neurology, or oncology were employed in private practice, whereas the opposite was true for LAIM. This likely reflects the expanding employment market in private practice for small animal practitioners compared with academia, and the comparably smaller number of large animal jobs in either setting. Only 58% of LAIM specialists reported that their scope of practice was limited to their field of training, suggesting that large animal internists often may be required to perform general practitioner or ambulatory work. As veterinary medicine continues to advance, it will be critical to highlight the value that an internist provides and foster a climate that will help LAIM Diplomates find fulfilling and well‐compensated employment.

These results objectively compare aspects of academic to private practice employment and provide quantitative information that may be a useful to those making or advising career decisions. Specialists employed in academia worked more hours per week, were less frequently employed part‐time, and reported lower annual incomes across all specialties with the exception of LAIM. Motivations for entering private vs academic practice were not considered in our study, but these 3 factors are likely relevant. Although income disparity is unlikely to change, academic positions that allow <100% appointment may be an attractive option for some diplomates. Although working hours were somewhat fewer per week in private practice, our study does not provide clarity regarding flexibility of work hours in academia. Most academic positions have dedicated off‐clinic time with the expectation of other academic pursuits such as research and teaching.

Almost one‐third of large animal internists were on call ≥15 nights per month, similar to the ACVS where over half of large animal surgeons in private practice reported as much on call responsibility.^1^ Although no significant difference was found, the proportions of diplomates in the small animal specialties who reported working ≥15 shifts per month on call were higher in private practice as compared with academia. Our observation that specialty section sizes were larger in academia likely contributes to this measurement, because higher numbers of diplomates will result in more capacity for sharing of the on‐call burden. Other benefits associated with a larger specialty group include opportunities for mentorship, peer learning, collaboration, coverage for time off, and social support. Ultimately, career path is a personal decision based on more factors than were considered in our study. Our findings, however, may be of value to some making career choices, or for administrators or managers considering how to make a working environment more attractive.

We employed the metrics of income, academic advancement, and practice ownership to estimate professional success among ACVIM Diplomates. Success is a term used loosely here, because the measures employed clearly do not reflect the personal values of all individuals surveyed. Still, all of these categories had significant disparities between men and women, with men being ahead in each instance. Incomes were 20% higher for men, overall, with a 9% and 29% income gap observed in academia and private practice, respectively. In the United States, the gender‐based income gap partially can be accounted for by factors such as education, employment status, hours worked, children, and others. Still, nearly 40% of the disparity can only be explained by gender itself.^9^ Although this was not measured, the same is likely true within the ACVIM. In our study, men in private practice worked approximately 3 hours/wk more than women; based on our estimated association between increased hours worked in private practice and increased income, this corresponds to a 2.5% increase in income. Although a contributing factor to the gap, it is not a primary explanation. The influence of gender is likely to manifest its effect on income through several pathways such as implicit and explicit biases in the workplace, differences in in negotiation strategies, career aspirations, and opportunities for sponsorship.^10^, ^11^, ^12^, ^13^ One critical factor that would be difficult to measure is the motivation to earn money between men and women. For example, only 47% of women, compared with 73% of men, responded that they were primary earners in their households, suggesting differences in financial responsibilities that may exist as a driving force for generating income.

Notably, the income gap percentages in the female‐dominated ACVIM are slightly higher than those observed in the male‐dominated ACVS, where an 18% overall income gap was observed, with 8% and 25% gaps reported in academia and private practice.^1^ Feminization of a profession has been shown to result in lower incomes.^14^ This outcome may be a result of a higher proportion of women earning lower incomes and driving average wages down, or already low average incomes subsequently attracting fewer men to the occupation. Similarly, within other occupations where women predominate, such as elementary education, nursing, and administrative assistants, women earn less than men.^15^ It may be that the changing demographics in veterinary medicine will neither improve the income gap nor the overall economics of the profession. Income for most specialists in private practice commonly is a function of production and these large disparities may, in part, reflect less revenue generated by female specialists, which would be a topic for consideration among practice owners as the profession continues to be feminized. The professional culture of practice could be structured around stereotypically masculine attitudes, behaviors, and schemas, and ultimately may not reward a female workforce in an equivalent manner. In light of the economics of an increasingly feminized profession and the data provided here, reconsidering compensation algorithms in ways that that reflect the behaviors of the current workforce should be considered.

Men achieved more career advancement as assessed by quantitative measurements of promotion rank and track, and by the proportion of those participating in practice ownership. This difference was no longer significant in the academic setting after controlling for age and year of diplomate status, but the same was not true regarding practice ownership. Certain forms of advancement are required in academia through promotion, yet practice ownership is more specifically a product of the intersection of a personal decision and an objective reward. In academia, the decision to move from associate to full professor is not mandatory but rather subject to individual choice, and twice the proportion of men reported full professor status compared with women. It is impossible to determine the extent to which these differences in advancement are a result of barriers, biases, or individual choice. Many examples of advancement gaps exist between men and women in academic settings; the gap observed in the ACVIM was quantitatively equal to that calculated between male and female ACVS Diplomates,^1^ and is similar to that seen between male and female physicians at US public medical schools^16^ and across higher education in general,^17^, ^18^ suggesting that barriers truly do exist for women. Twice the proportion of men in the ACVIM participated in practice ownership as compared with women, a contrast mirrored among ACVS Diplomates,^1^ and reflected in a broader statistic indicating that men are almost twice as likely to start a new business in the United States as compared with women.^19^ This finding, too, suggests some effects of the common thread of gender, such as differences in opportunities, enthusiasm, or preparedness for business ownership and management.

Predictably, balancing a personal life with one's career is challenging for most diplomates. This is expected for many clinical jobs, yet it remains influential when choosing a profession. Most diplomates are driven by a passion for what they do, and were either somewhat satisfied or better with their career. Especially among the younger millennial or generation Y demographic, focusing on family and leisure and workplace flexibility improves workplace retention.^20^, ^21^, ^22^ Conversely, long hours and irregular schedules, which undoubtedly affect the of climate of the veterinary profession, decrease satisfaction and cause people to leave their jobs.^21^ In the competitive hiring environment for ACVIM specialists, it may be useful to consider that many people value flexibility and culture over income, and structuring the workplace to foster these ideals may be attractive for potential employees.

Despite our objective findings highlighting measurable differences in how men and women experience their professional lives, it appears a relative underestimation of these variations exists. Overall, men appeared to be less aware than women about gender inequality, although nearly half of male respondents did indicate they believed it existed. This observation is consistent with previous findings regarding how men and women perceive persistent biases within the profession.^1^, ^10^ The subjective line of questioning delineated in Figure 5 regarding the impact of one's own gender on salary shows that some men are aware they may experience an advantage, and over one‐third of women suspect they are at some disadvantage. These responses reflect prevailing beliefs among diplomates, and emphasize the need to increase awareness of how implicit biases and discrepancies have repercussions in the workplace. Dissemination of information reflecting both the objective and subjective experiences of ACVIM Diplomates can increase awareness, and promote more equitable environments.

One limitation of our study was the paucity of information regarding individuals from underrepresented minority groups. The lack of diversity within the ACVIM is reflective of historical trends in US veterinary medical programs, which were >90% white until the late 2010s when concerted diversity initiatives were able to increase groups traditionally underrepresented in veterinary medicine to almost 20%.^23^ Similarly, our study was not able to report findings for other groups that might experience bias or discrimination in the workplace, such as the lesbian‐gay‐bisexual‐transgender‐queer‐intersex‐asexual community, or those identifying as nonbinary, because of very small response numbers or lack of identifying information. As is true with any survey study, it is impossible to determine the effects of response bias, or the extent to which respondents answered truthfully and accurately. Finally, although comparisons can be made quantitatively and statistically here, these comparisons should be interpreted as correlations, and not causative relationships. Although repeatable correlations suggest that women experience their careers differently than men, it would be unfair to suggest that gender itself is the effector. The element of individual choice and other unmeasurable factors cannot be accounted for in our study, and is of tremendous importance when considering data that so broadly evaluates personal and professional lives. Overall, Diplomates of the ACVIM are a highly educated, successful, and satisfied group with a diversity of professional paths and opportunities, making a career in the field and its specialties an attractive option to young veterinarians.

## CONFLICT OF INTEREST DECLARATION

Authors declare no conflict of interest.

## OFF‐LABEL ANTIMICROBIAL DECLARATION

Authors declare no off‐label use of antimicrobials.

## INSTITUTIONAL ANIMAL CARE AND USE COMMITTEE (IACUC) OR OTHER APPROVAL DECLARATION

Institutional Review Board at the University of Wisconsin‐Madison waived formal review of the protocol due to the nature of the study in accordance with Federal Regulation 45 CFR 46.102(d).

## HUMAN ETHICS APPROVAL DECLARATION

Authors declare human ethics approval was not needed for this study.

## Supporting information


**Supplementary Item 1:** • • •

**Supplementary Item 2:** • • •
