# Supplementary material for: Work, life, and the gender effect: Perspectives of ACVIM Diplomates in 2017. Part 1—Specialty demographics and measures of professional achievement
Source: J Vet Intern Med. 2020 Aug 17;34(5):1825–36. doi: 10.1111/jvim.15872 (PMC7517495; doi:10.1111/jvim.15872)
Supplement: Supplementary file 1 — Supplementary Item 1: • • • [file JVIM-34-1825-s001.pdf]

ACVIM Career-Life Balance Survey

In what specialty area was your residency training completed?
- ❑ Small Animal Internal Medicine
- ❑ Large Animal Internal Medicine (Mixed Equine and Farm Animal)
- ❑ Equine Internal Medicine
- ❑ Farm Animal Medicine
- ❑ Cardiology
- ❑ Oncology
- ❑ Neurology/Neurosurgery

In what year did you achieve Diplomate status for the specialty in which you are currently practicing?
(1970-2015)

What is the status of your current employment?
- ❍ Employed, full-time
- ❍ Employed, part-time
- ❍ Unemployed

Are you currently practicing clinical veterinary medicine?
- ❍ Yes
- ❍ No

Is your current scope of practice limited to the specialty field in which you were trained? (i.e. internal medicine vs. general practice)
- ❍ Yes
- ❍ No

What is the primary species of your current employment?
- ❍ Small Animal
- ❍ Equine
- ❍ Large Animal (Equine and Food Animal)
- ❍ Food Animal Only
- ❍ Mixed (Large and Small)
- ❍ Laboratory Animal
- ❍ Industry/Research (not animal based)
- ❍ I am not currently practicing veterinary medicine
- ❍ Other ____________________

Are you currently practicing on the species for which you trained during your residency?
- ❍ Yes
- ❍ No, I completed a large animal medicine residency and currently practice on small animals
- ❍ No, I completed a small animal medicine residency and currently practice on large animals
- ❍ No, I completed a residency and now work in industry/research with an emphasis on my specialty area
- ❍ Other ____________________

Please expand upon your decision to change your specialty from your area of residency training

Type of practice in which you are currently employed:
- ❍ Academia
- ❍ Private Practice (Specialty)
- ❍ General Practice
- ❍ Industry
- ❍ Military
- ❍ Other ____________________

Academic employment: current job title
- ❍ Clinical Instructor/Lecturer
- ❍ Assistant Professor (tenure track)
- ❍ Assistant Professor (clinical track)
- ❍ Associate Professor (tenured)
- ❍ Associate Professor (clinical)
- ❍ Professor (full, tenured)
- ❍ Clinical Professor (non-tenured)
- ❍ Dean/Associate Dean
- ❍ Other Administrative Role ____________________
- ❍ Other ____________________

Private Practice, General Practice: current job title
- ❍ Associate Veterinarian
- ❍ Practice Owner (shareholder,
- ❍ Practice Owner (shareholder, >50%)
- ❍ Freelance/locum
- ❍ Other ____________________

Industry: current job description
- ❍ Scientist/Researcher
- ❍ Salesperson
- ❍ Management/Consultant
- ❍ Executive
- ❍ Other ____________________

Please provide your current job title:

Please provide your current job title:

How many jobs practicing clinical veterinary medicine have you held since finishing your residency program (including your current position)?

❍ 0
❍ 1
❍ 2
❍ 3
❍ 4
❍ 5
❍ 6+

How many ACVIM diplomates does your practice or institution employ in each of the following subspecialties (for those in mixed practice or academia, you may answer for both large and small animal specialties)

______ Small Animal Internal Medicine
______ Large Animal Internal Medicine
______ Oncology (Medical and Radiation)
______ Cardiology
______ Neurology

How many <u>female</u> ACVIM diplomates does your practice or institution employ in each of the following subspecialties (for those in mixed practice or academia, you may answer for both large and small animal specialties)

______ Small Animal Internal Medicine
______ Large Animal Internal Medicine
______ Oncology (Medical and Radiation)
______ Cardiology
______ Neurology

How many <u>male</u> ACVIM diplomates does your practice or institution employ in each of the following subspecialties (for those in mixed practice or academia, you may answer for both large and small animal specialties)

______ Small Animal Internal Medicine
______ Large Animal Internal Medicine
______ Oncology (Medical and Radiation)
______ Cardiology
______ Neurology

On average, how many total hours per week do you work?
❍
❍ 40-49 hours
❍ 50-59 hours
❍ 60+ hours

On average, how many nights/weekend days per month are you on call (primary or backup)?
❍ 0
❍ 1-3
❍ 4-7
❍ 7-10
❍ 10-14
❍ 15+

Please rank the following in order of how influential these factors were in your decision to pursue your current professional position (i.e.. academia vs. private practice, surgeon vs. general practitioner etc). Please rank from MOST influential (1) to LEAST influential (9)
_______ Financial compensation
_______ Quality of work/life balance
_______ Location
_______ Number of hours of work required
_______ Emergency responsibilities
_______ Benefits (i.e.. health insurance, pension etc.)
_______ Passion for the job
_______ Ability to do research
_______ Opportunity for teaching

How satisfied are you currently with your career?
❍ Very Satisfied
❍ Satisfied
❍ Somewhat Satisfied
❍ Neutral
❍ Somewhat Dissatisfied
❍ Dissatisfied
❍ Very Dissatisfied

Current relationship status
❍ Single (never married)
❍ Married/Domestic Partnership
❍ Separated
❍ Divorced
❍ Widow/Widower

How many adults currently live in your home?
- ❍ 1
- ❍ 2
- ❍ 3
- ❍ more than 3

Using the sliding scale below, please indicate your personal income during the past 12 months, before taxes (for salaries greater than or less than indicated on the sliding scale, please select the highest or lowest option, respectively).
______ Personal Income (in Thousands)

Are you the primary earner in your household? (if you are the only member of your household, please answer "Yes")
- ❍ Yes
- ❍ No
- ❍ Our incomes are relatively equivalent

Is your partner/spouse also a veterinarian?
- ❍ Yes
- ❍ No
- ❍ NA

Do you have children?
- ❍ Yes
- ❍ No

If you do not have children:
- ❍ I did not or do not plan to have children
- ❍ I have not attempted to have children, but hope to have children in the future
- ❍ I attempted to have biologic children, but I and/or my parter were unable to

How many children do you have?
- ❍ 1
- ❍ 2
- ❍ 3
- ❍ 4
- ❍ 5
- ❍ 6
- ❍ 7
- ❍ 8+

Using the sliding scale below, please indicate the ages of your oldest and, if applicable, youngest child (please use a value of 1 for any child under the age of 1 year).
______ Oldest Child
______ Youngest Child

Do one or more of your children currently live with you?
❍ No
❍ Yes, full time
❍ Yes, part time

If one or more of your children are under the age of 13, do you require childcare?
❍ Yes
❍ No, I stay home with them
❍ No, my partner/spouse stays home with them
❍ My partner and I share this responsibility

If one or more of your children are older than 13, did you require childcare before they were 13?
❍ Yes
❍ No, I stayed home with them
❍ No, my partner/spouse stayed home with them
❍ My partner and I shared this responsibility

Outside scheduled work hours, who is the primary caretaker for your children?
❍ I am
❍ My partner/spouse is
❍ My partner/spouse and I share this responsibility equally
❍ A family member who is neither me, nor my partner/spouse
❍ Other (day care, nanny, etc.)
❍ My children are no longer under my care

Using the sliding scale, please indicate how old you were when you had, or adopted, or provided foster care for your first child.
______ Age, years

Using the sliding scale, please indicate how old were when you had, or adopted, or provided foster care for your youngest child?
______ Age, years

If you currently are, or have been, married or in a committed relationship, do you feel that your career has:
❍ had a positive impact on your relationship/s
❍ had a negative impact on your relationship/s
❍ neutral - no overall positive or negative impact on your relationship/s

Please select your level of agreement or disagreement with the following statement:<div>The decision to pursue my career did/will affect my decision or ability to pursue having children.
<div>
</div></div>

- ❍ Strongly Disagree
- ❍ Disagree
- ❍ Neither Agree nor Disagree
- ❍ Agree
- ❍ Strongly Agree

Do you feel that having a family has had an impact on your career?

- ❍ Yes, having a family has positively affected my career development
- ❍ Yes, having a family has negatively affected my career development
- ❍ No, having a family has not had an overall positive or negative impact on my career development

Please elaborate on your previous answer:

Do you feel that having a family will impact/would have impacted your career?

- ❍ Yes, having a family will/would have positive affected my career development
- ❍ Yes, having a family will/would have negatively affected my career development
- ❍ No, having a family will not/would not have had an overall positive or negative impact on my career development

Please elaborate on your previous answer:

Please select your level of agreement or disagreement with the following statement: <div>Maintaining my professional occupation has had a negative impact on my ability to participate in my family life, especially as it refers to raising children.</div>

- ❍ Strongly disagree
- ❍ Somewhat disagree
- ❍ Neither agree nor disagree
- ❍ Somewhat agree
- ❍ Strongly agree

Please select your level of agreement or disagreement with the following statement: <div>Maintaining my professional career has had a negative impact on my ability to pursue interests outside of work</div>

- ❍ Strongly agree
- ❍ Somewhat agree
- ❍ Neither agree nor disagree
- ❍ Somewhat disagree
- ❍ Strongly disagree

If so, how?

When during your career did you/do you plan to have or adopt your first child?
- ❍ Prior to Veterinary School
- ❍ Veterinary School
- ❍ Internship
- ❍ Residency
- ❍ Other graduate training
- ❍ Within the first 5 years of full-time work
- ❍ After the first 5 years of full-time work
- ❍ Other ____________________
- ❍ I do not/did not plan on having children

If you have more than one child, when during your career did you have or adopt your youngest child?
- ❍ I only have one child
- ❍ Prior to Veterinary School
- ❍ Veterinary school
- ❍ Internship
- ❍ Residency
- ❍ Other graduate training
- ❍ Within the first 5 years of full-time work
- ❍ After the first 5 years of full-time work
- ❍ Other ____________________

Please select your level of agreement or disagreement with the following statement: <div>The stage of your career (i.e. training vs. employment, number of years practicing post residency) played/will play an important role in family planning for you and your significant other. </div>
- ❍ Strongly Disagree
- ❍ Disagree
- ❍ Neither Agree nor Disagree
- ❍ Agree
- ❍ Strongly Agree
- ❍ I do not plan on having children

Has having children affected the type of practice or career path you have chosen? (i.e. private practice vs. academia, small animal vs. large animal, associate veterinarian vs. practice owner, surgical practice vs. general practice)
- ❍ Yes
- ❍ No

If so, how?

Please select your level of agreement or disagreement with the following statement:<div>Having children has, or may in the future, influence me to pursue a career path outside clinical veterinary practice.</div>

- ❍ Strongly Disagree
- ❍ Disagree
- ❍ Neither Agree nor Disagree
- ❍ Agree
- ❍ Strongly Agree
- ❍ I do not plan on having children

Please select your level of agreement or disagreement with the following statement: <div>Having children has, or may in the future, influence me to decrease the number of hours worked per week in my job </div>

- ❍ No, my hours are/will remain the same
- ❍ No, my working hours have/will increase
- ❍ Yes, my working hours have/will have decreased but I still work full time (40+ hours)
- ❍ Yes, my working hours have/will have decreased to 20-40 hours/week
- ❍ Yes, my working hours have/will have decreased to
- ❍ I do not plan on having children

If so, please elaborate:

Using the sliding scale below, please indicate how many weeks of parental leave you took. If you have had more than one child, please list the most time you took off, and the least time you took off. If you took off more than a year, please indicate 53 weeks.

______ Weeks (most time, or only child)
______ Weeks (least time)

Does your employer offer parental leave that is:

- ❍ Fully paid
- ❍ Unpaid
- ❍ Partially compensated

Was the paid portion of your parental leave covered by (check all that apply):

- ❑ Vacation Time
- ❑ Sick Leave
- ❑ Short-term disability/ Income Continuation (or the equivalent)
- ❑ Compensation that is specifically designated for parental leave

Using the sliding scale below, please indicate how many weeks of paid parental leave you received, and how many weeks of unpaid or partially paid parental leave you received (if you have had more than one child, please indicate the most amount of paid or unpaid leave).
______ Weeks of Paid Leave
______ Weeks of Unpaid Leave
______ Weeks of Partially Paid Leave

Please select your level of agreement or disagreement with the following statement:<div>The amount of time allowed for parental leave from my job was adequate.</div>
- Strongly Disagree
- Disagree
- Neither Agree nor Disagree
- Agree
- Strongly Agree

Please select your level of agreement or disagreement with the following statement:<div>My boss/supervisor was supportive of the time I took for parental leave.</div>
- Strongly Disagree
- Disagree
- Neither Agree nor Disagree
- Agree
- Strongly Agree
- I did not take parental leave

Please select your level of agreement or disagreement with the following statement: <div>My colleagues were supportive of the time I took for parental leave. </div>
- Strongly Disagree
- Disagree
- Neither Agree nor Disagree
- Agree
- Strongly Agree
- I did not take parental leave

Please select your level of agreement or disagreement with the following statement: <div>Taking time for parental leave affected my ability to reach my career goals.</div>
- Strongly Disagree
- Disagree
- Neither Agree nor Disagree
- Agree
- Strongly Agree
- I did not take parental leave

Do you feel that your significant other has a career/job that allows for great flexibility, as relates to time required doing their job?

- ❍ Yes, my significant other stays at home full time
- ❍ Yes, my significant other has a job that allows for significant time off and/or part time work
- ❍ Yes, my significant other works from home
- ❍ Yes, my significant other has more flexibility with their hours than I do
- ❍ No, my significant other and I have equally demanding jobs
- ❍ No, my significant other has a more demanding schedule than mine

Do you feel that your gender has had an impact on your salary?

- ❍ No, my salary is equal to that of my opposite-gender, but similarly qualified colleagues
- ❍ Yes, my salary is greater than my opposite-gender, but similarly qualified colleagues
- ❍ Yes, my salary is less than my opposite-gender, but similarly qualified colleagues
- ❍ Unsure - I do not know how my salary compares with that of my opposite-gender, but similarly qualified colleagues

Please elaborate:

Do you feel that your gender has influenced your ability to secure a desired job position?

- ❍ No, my gender did not have an impact on my ability to acquire or retain the job I desired
- ❍ Yes, my gender had a positive impact on my ability to acquire or retain the job I desired
- ❍ Yes, my gender had a negative impact on my ability to acquire or retain the job I desired
- ❍ Unsure - I do not know if gender was a factor in my ability to acquire or retain my desired job

Do you feel that your gender has affected your ability to be promoted?

- ❍ No
- ❍ Yes, negatively
- ❍ Yes, positively
- ❍ Unsure

Do you feel that your gender has affected your current job responsibilities? If yes, please explain

- ❍ Yes
- ❍ No
- ❍ Unsure

If so, how?

Do you feel that your gender affects your interaction with clients? If so, please explain

- ❍ Yes
- ❍ No
- ❍ Unsure

If so, how?

Apart from your own experiences, do you feel that gender inequality exists for internists/specialists in private practice?

- ❍ Definitely yes
- ❍ Probably yes
- ❍ Maybe
- ❍ Probably not
- ❍ Definitely not

Apart from your own experience, do you feel that gender inequality exists for internists/specialists in academic practice?

- ❍ Definitely yes
- ❍ Probably yes
- ❍ Maybe
- ❍ Probably not
- ❍ Definitely not

Has anyone (client, colleague, co-worker etc.) made a comment about your gender as it relates to career performance, potential, or productivity? (i.e. a potential assumption, generalization, or stereotype?)

- ❍ Yes
- ❍ No

Please elaborate on how you feel this has occurred

Please select your level of agreement with the following statement:<div>Overall, I feel I have achieved a balance between having a satisfying career and a satisfying personal life. </div>

- ❍ Strongly agree
- ❍ Somewhat agree
- ❍ Neither agree nor disagree
- ❍ Somewhat disagree
- ❍ Strongly disagree

How old are you?
- 25-30
- 31-35
- 36-40
- 41-45
- 46-50
- 51-55
- 56-60
- 61-65
- 66-70
- 71+

With which gender do you identify?
- Male
- Female
- Other

Race/Ethnicity
- White
- Black or African American
- Hispanic or Latino
- Asian or Pacific Islander
- Native American or American Indian
- Other ____________________

In what part of the world do you currently reside
- United States of America
- Canada
- Central America
- South America
- Europe
- Asia
- Australia/New Zealand
- Africa

Which region of the country do you currently live?
- ❍ Midwest - IA, IL, IN, KS, MI, MN, MO, ND, NE, OH, SD, WI
- ❍ Northeast - CT, DC, DE, MA, MD, ME, NH, NJ, NY, PA, RI, VT
- ❍ Southeast - AL, AR, FL, GA, KY, LA, MS, NC, SC, TN, VA, WV
- ❍ Southwest - AZ, NM, OK, TX
- ❍ West - CA, CO, ID, MT, NV, OR, UT, WA, WY
- ❍ Hawaii
- ❍ Alaska
- ❍ Puerto Rico

Outside of your DVM and DACVIM, what other degree/s have you earned?
- ❑ MS
- ❑ PhD
- ❑ Other board certification ____________________
- ❑ MBA
- ❑ JD
- ❑ Other ____________________
- ❑ None
