# Supplementary material for: Work, life, and the gender effect: Perspectives of ACVIM Diplomates in 2017. Part 1—Specialty demographics and measures of professional achievement
Source: J Vet Intern Med. 2020 Aug 17;34(5):1825–36. doi: 10.1111/jvim.15872 (PMC7517495; doi:10.1111/jvim.15872)
Supplement: Supplementary file 2 — Supplementary Item 2: • • • [file JVIM-34-1825-s002.pdf]

Email Subject title: Participation in ACVIM Work-Life Balance Study

Dear Diplomate,

This survey is being sent out to ACVIM diplomates with the purpose of identifying trends among veterinary internists and specialists of the college (Oncology, Neurology, Cardiology) in the United States and abroad. Our aims are to assess the relationship between the careers of practicing specialists and their personal lives, and to create an objective dataset that summarizes the current landscape of a career in internal medicine or one of the sub-specialties. In light of the changing gender demographic in veterinary medicine, this is an opportunity to identify some of the challenges that men and women face in balancing a successful career with personal time and, those that women, specifically, may face professionally.

While this topic has been extensively studied in the field of human medicine, investigation in the veterinary medical community has been limited. We have previously administered this survey to diplomates of the ACVS. The survey was well received, and a portion of the results were presented as a Keynote address at the 2015 ACVS Surgery Summit. A manuscript is in preparation for publication. Our goal is to present and publish the data from this survey with the ACVIM in a similar manner.

The project is not associated intellectually or financially with the American College of Veterinary Internal Medicine (ACVIM). The content is solely the responsibility of the authors and does not represent the official views of the ACVIM.

The survey will take approximately 10-15 minutes to complete. Answering this survey is completely elective, and you may choose to stop participating at any time, even after beginning the survey, and may skip any question you'd like. Responses are anonymous; answers cannot be linked back to individuals. Please do not include your name or other identifying information.

To participate, please click the link provided below, or copy and paste into your browser:

We urge you to take the time to fill out the survey and answer the questions for the benefit of the ACVIM organization and the veterinary community at large.

If you have any questions, comments, or concerns, please feel free to contact us at this email address: XXXXXX@XXXXX.edu

Thank you for your participation.
